# The Histidine Kinase AHK5 Integrates Endogenous and Environmental Signals in *Arabidopsis* Guard Cells

**DOI:** 10.1371/journal.pone.0002491

**Published:** 2008-06-18

**Authors:** Radhika Desikan, Jakub Horák, Christina Chaban, Virtudes Mira-Rodado, Janika Witthöft, Kirstin Elgass, Christopher Grefen, Man-Kim Cheung, Alfred J. Meixner, Richard Hooley, Steven John Neill, John Travers Hancock, Klaus Harter

**Affiliations:** 1 Division of Biology, Imperial College London, London, United Kingdom; 2 Zentrum für Molekularbiologie der Pflanzen / Pflanzenphysiologie, Universität Tübingen, Tübingen, Germany; 3 Department of Nano Optics, Institute for Physical and Theoretical Chemistry, Universität Tübingen, Tübingen, Germany; 4 Department of Biology and Biochemistry, University of Bath, Bath, United Kingdom; 5 Centre for Research in Plant Science, University of the West of England, Bristol, United Kingdom; Umeå Plant Science Centre, Sweden

## Abstract

**Background:**

Stomatal guard cells monitor and respond to environmental and endogenous signals such that the stomatal aperture is continually optimised for water use efficiency. A key signalling molecule produced in guard cells in response to plant hormones, light, carbon dioxide and pathogen-derived signals is hydrogen peroxide (H_2_O_2_). The mechanisms by which H_2_O_2_ integrates multiple signals via specific signalling pathways leading to stomatal closure is not known.

**Principal Findings:**

Here, we identify a pathway by which H_2_O_2_, derived from endogenous and environmental stimuli, is sensed and transduced to effect stomatal closure. Histidine kinases (HK) are part of two-component signal transduction systems that act to integrate environmental stimuli into a cellular response via a phosphotransfer relay mechanism. There is little known about the function of the HK AHK5 in *Arabidopsis thaliana.* Here we report that in addition to the predicted cytoplasmic localisation of this protein, AHK5 also appears to co-localise to the plasma membrane. Although *AHK5* is expressed at low levels in guard cells, we identify a unique role for AHK5 in stomatal signalling. *Arabidopsis* mutants lacking AHK5 show reduced stomatal closure in response to H_2_O_2_, which is reversed by complementation with the wild type gene. Over-expression of AHK5 results in constitutively less stomatal closure. Abiotic stimuli that generate endogenous H_2_O_2_, such as darkness, nitric oxide and the phytohormone ethylene, also show reduced stomatal closure in the *ahk5* mutants. However, ABA caused closure, dark adaptation induced H_2_O_2_ production and H_2_O_2_ induced NO synthesis in mutants. Treatment with the bacterial pathogen associated molecular pattern (PAMP) flagellin, but not elf peptide, also exhibited reduced stomatal closure and H_2_O_2_ generation in *ahk5* mutants.

**Significance:**

Our findings identify an integral signalling function for AHK5 that acts to integrate multiple signals via H_2_O_2_ homeostasis and is independent of ABA signalling in guard cells.

## Introduction

Plants are constantly exposed to a large multitude of environmental stimuli, and under adverse conditions, are mostly able to survive due to their ability to sense and transduce these signals into cellular and physiological responses. Hydrogen peroxide (H_2_O_2_) is a form of reactive oxygen species (ROS) generated by plants via several mechanisms, which include metabolic processes such as respiration and photosynthesis as well as reactions to environmental stimuli such as water deficit, high and low temperature, pollutants, UV-B light and pathogen challenge [1]. It is now well accepted that controlled or regulated production of H_2_O_2_ is beneficial to the plant. H_2_O_2_ acts as a signal and/or second messenger enabling the plant to activate physiological processes resulting in protection from and adaptation to environmental stress [2].

H_2_O_2_ regulates a number of molecular and cellular processes in plants ranging from gene expression, programmed cell death, cell division, elongation growth, and stomatal closure [3]. The molecular mechanisms by which each of these processes occurs through H_2_O_2_ signalling have not been fully clarified. Recently, several targets for H_2_O_2_ have been identified in *Arabidopsis*, including protein kinases and phosphatases [4]–[8]. In relation to stomatal closure and redox signalling, the ABI1 and ABI2 members of the protein phosphatase 2C family are redox regulated in response to ABA [9], [10]. Moreover, the MAP kinase MPK3 was shown recently to be essential for both ABA and H_2_O_2_-inhibition of stomatal opening in *Arabidopsis* [11]. The protein kinase OST1 regulates H_2_O_2_ production in guard cells through signalling pathways requiring the ROS-producing NADPH oxidase subfamily of proteins (namely, AtRBOHD and AtRBOHF [12], [13]). Thus, reversible protein phosphorylation appears to be a key mechanism by which cellular responses to multiple stimuli are regulated via H_2_O_2_ in guard cells and other cell types.

An alternative mechanism by which H_2_O_2_ acts on proteins is by oxidation of Cys residues [14]. H_2_O_2_ oxidation of –SH groups on Cys residues in proteins causes either disulfide bond formation or the formation of sulfenic acid groups. The latter can be sequentially oxidised to sulfinic and sulfonic acid groups at higher concentrations of H_2_O_2_. Reversal of oxidation occurs under reducing conditions, for example, by reduced glutathione or thioredoxin [14]. Until recently, there was little evidence that H_2_O_2_ action on Cys residues is responsible for defined physiological responses. In recent work, we have shown that one member of the *Arabidopsis* hybrid histidine kinase (HK) family, the ethylene receptor ETR1, is a potential target for H_2_O_2_ during stomatal closure [15]. ETR1 is required for H_2_O_2_-mediated stomatal closure, with the Cys65 residue of ETR1 being essential. Intriguingly the HK domain of ETR1 was not required for H_2_O_2_-induced closure [15]. In recent developments we have also shown that ETR1 has a dual function in guard cells, that of perceiving ethylene as well as acting as a target for H_2_O_2_, thereby mediating downstream signalling processes to initiate stomatal closure [16].

The hybrid HK family of receptor proteins are part of the two-component signal perception and transduction system in plants [17]. Perception of a signal by a hybrid HK leads to autophosphorylation of a His residue in the HK domain, followed by a phosphotransfer reaction to an Asp residue on its receiver domain. Subsequently, a relay of the phosphoryl residue occurs to a His residue on a histidine phosphotransfer protein (HP) followed by phosphorylation of an Asp residue on a response regulator (RR) protein [18]. Representative HK family members in plants are the cytokinin and ethylene receptors [17]. However, the plant's two-component signalling network appears to contribute to several other signal response pathways. For instance, recent work has demonstrated functional cross-talk between cytokinin and light (phytochrome B) signalling [19], [20]. These overlaps in signalling processes induced by multiple stimuli suggest that two-component proteins are key sensors and transducers of various environmental and endogenous signals.

One mechanism by which different signalling processes and pathways may be integrated is *via* common second messenger molecules. ROS such as H_2_O_2_ are ideal candidates for such messenger molecules acting as focal points for cross-talk between a wide array of signalling cascades [3]. This is evidenced by a large overlap in the expression of genes that are regulated by ROS on the one hand and by environmental stimuli on the other. For example, H_2_O_2_-regulated genes are also regulated by drought, cold, UV-B light and pathogen attack [21], [22] as well as by ABA [23]. Clearly, there exist multiple targets for H_2_O_2_ to mediate its effects in specific cells and tissues. Functional overlap is also likely to exist between these pathways, with certain targets acting as integrators of multiple stimuli.

In an attempt to identify further targets for H_2_O_2_ signalling in guard cells, the function of a least-characterised member of the HK family in *Arabidopsis*, namely AHK5, was investigated. AHK5 is predicted to be the only cytoplasmic HK, with both a canonical HK domain and receiver domain, classifying it as a hybrid HK [17]. Our data here indicate that AHK5 also co-localises to the plasma membrane. Recent work has identified a function for AHK5 in counteracting the ethylene and ABA-regulated growth response in *Arabidopsis* roots [24]. However its role in integrating signalling responses to H_2_O_2_ is not known.

Using a combination of molecular genetic, cell imaging, biochemical and physiological tools, we show that AHK5 is a key player in H_2_O_2_ homeostasis in *Arabidopsis* guard cells in response to environmental and endogenous signals, including NO, ethylene, darkness and bacterial flagellin. Intriguingly, AHK5 does not appear to be involved in the ABA signalling pathway in stomata. Our data suggest a regulatory function of AHK5 that is essential for guard cell response to abiotic and biotic environmental stimuli.

## Results

### Intracellular localisation and tissue-specific expression of AHK5

**Figure 1:**
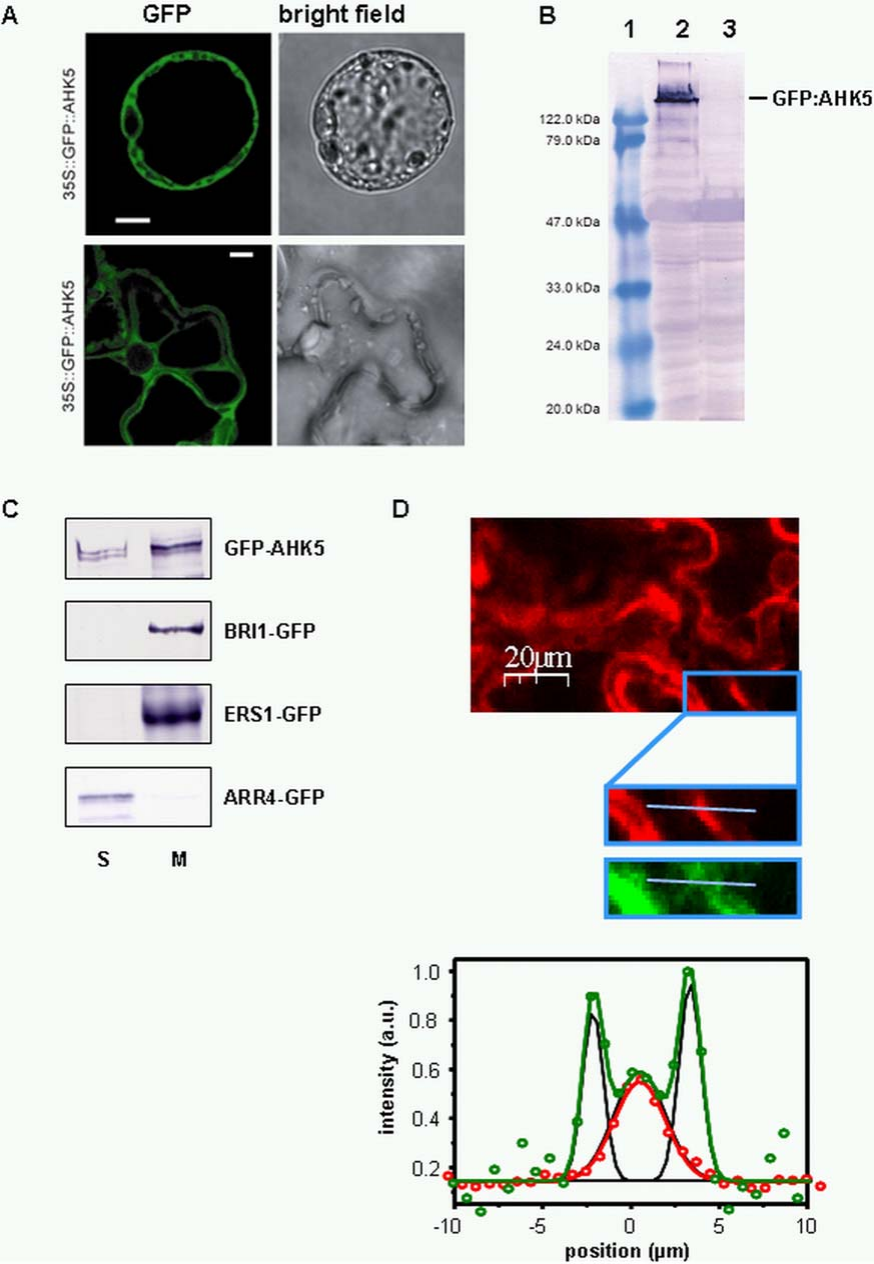
Subcellular localisation of AHK5 in plant cells. (A) Confocal images of *Arabidopsis* protoplasts and tobacco (*Nicotiana benthamiana*) leaf cells transiently transformed with a construct expressing *P_35S_:GFP-AHK5* cDNA. Left panel, GFP fluorescence; right panel, bright field image. The bars represent 10 μm. (B) Western blot showing the expression of full-length GFP-AHK5 in transiently transformed tobacco leaf cells using anti-GFP antibody. Lane 1, protein standard; lane 2, extracts from cells transformed with a *P_35S_-GFP-AHK5* construct; lane 3, extracts from cells transformed with the empty vector. (C) Cell fractionation of transiently transformed tobacco leaf cells expressing either GFP-AHK5, the microsomal marker BRI-GFP, the ER marker ERS1-GFP or the soluble marker ARR4-GFP.Two days after the infiltration of the *Agrobacteria* the leaf tissue was harvested and total protein extracted. The microsomal fraction (M) and the soluble fraction (S) were separated by ultracentrifugation. Equal cell equivalents were loaded per lane. (D) Fluorescence intensity images (upper panel) and the corresponding intensity profiles (lower diagram) of the indicated plasmalemma-cell wall section (blue bar in the magnification) of two adjacent, transiently transformed tobacco leaf cells co-expressing GFP-AHK5 (green dots) and the plasma membrane marker pm-rk-CD3-1007 (red dots). The red line represents the mono-peak Gauss fit of RFP fluorescence and the green line the multi-peak Gauss fit of GFP fluorescence (green). The single fits which compose the multi-peak Gauss fit of GFP, are shown in black.

AHK5 is predicted to be the only cytoplasmic HK amongst the canonical HK class of proteins in *Arabidopsis* [17]. In order to confirm this, we performed *in vivo* localisation studies using 35S promoter-driven GFP fusion constructs of *AHK5*. In transiently transformed *Arabidopsis* protoplasts and tobacco (*Nicotiana benthamiana*) leaf cells the full-length fusion protein was expressed and was present in the cytoplasm, independent of whether the GFP tag was fused to the N-terminus or C-terminus of AHK5 (Fig. 1A and B, and Data S1). To substantiate our cell biological results we also performed cell fractionation experiments with extracts from transiently transformed tobacco leaf cells expressing *35S:GFP-AHK5* or several GFP-marker protein fusion genes. Whereas the ER marker ERS1-GFP [25] and the plasmalemma/endosome marker BRI1-GFP [26] were detected in the microsomal fraction and the cytoplasmic/nucleoplasmic marker ARR4-GFP [19] in the soluble fraction, GFP-AHK5 was found in both fractions (Fig. 1C). This intracellular distribution could be substantiated by recording the wavelength-specific intensity distribution of a cytoplasm-plasmalemma-cell wall section of neighbouring tobacco epidermal cells co-expressing GFP-AHK5 and the red fluorescent plasmalemma marker pm-rk-CD3-1007 (Fig. 1D; [27]). Whereas pm-rk-CD3-1007 showed one distinct peak representing the two plasmalemmata of the adjacent cells, three peaks were observed for GFP-AHK5 (Fig. 1D). The medial GFP-AHK5 peak showed a perfect overlay with pm-rk-CD3-1007, whereas the other two peaks extended to the cytoplasmic sites of the adjacent cells. Our results therefore suggest that AHK5 is a HK that is localised both in the cytoplasm and at the plasmalemma of plant cells.

**Figure 2:**
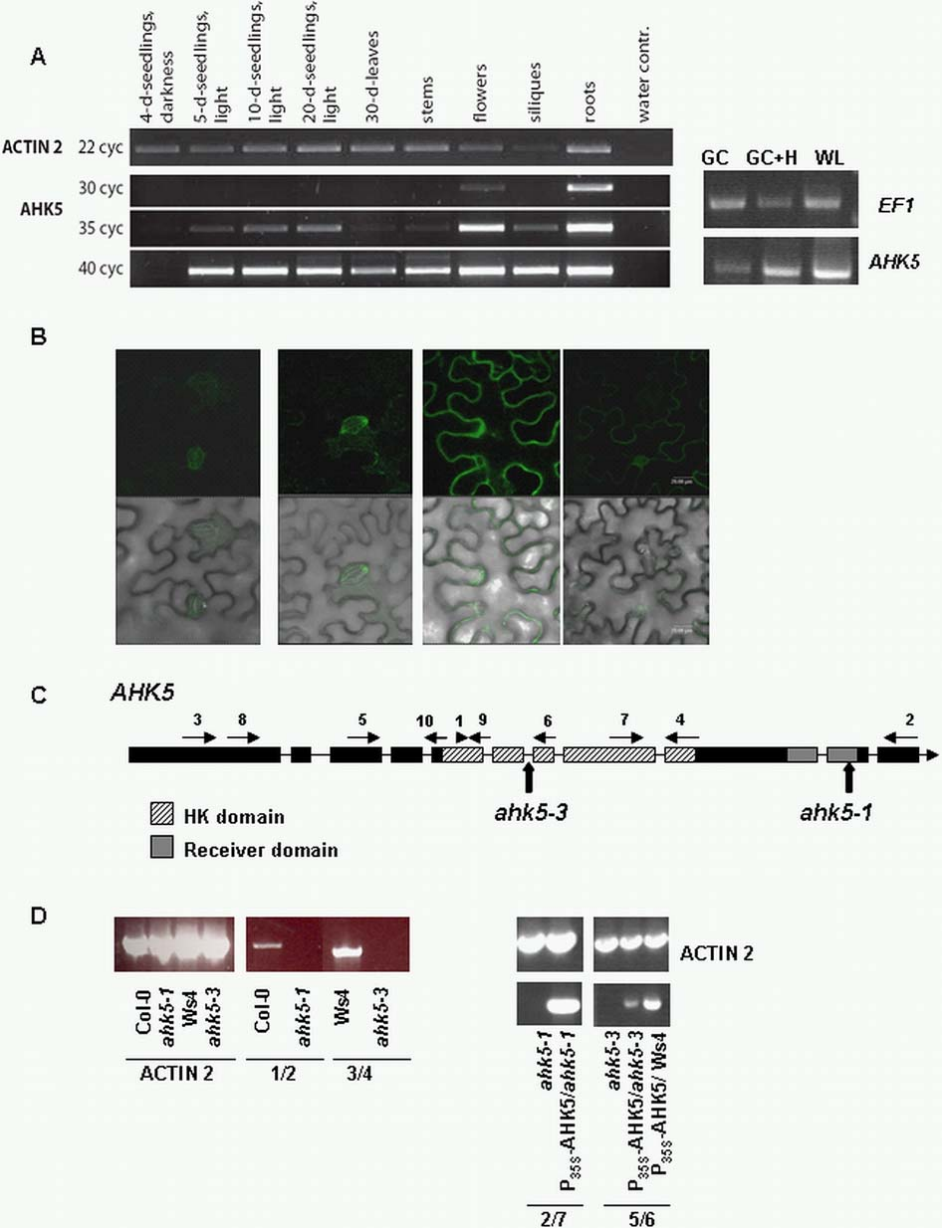
Expression pattern of *AHK5* and characterisation of the T-DNA insertion sites in *ahk5-1* and *ahk5-3*. (A) Steady-state levels of *AHK5* transcript in different tissues and developmental stages of *Arabidopsis* detected by semi-quantitative RT-PCR using different cycle numbers. For the detection of *AHK5*, up to 40 cycles of PCR were performed using primers 3 and 10 (left panel) or primers 8 and 9 and 40 cycles of PCR (right panel). GC, cDNA from guard cell-enriched non-treated leaf sample; GC+H, cDNA from guard cell-enriched leaf samples treated with 0.5 mM H_2_O_2_ for 2 h; WL, cDNA from whole-leaf sample. ACTIN and EF1 were used as controls. Primer numbers indicated in panel C. (B) Expression of a *P_AHK5_-GFP-AHK5genomic* construct in transiently transformed tobacco leaf cells. *Agrobacteria* carrying the *P_AHK5_-GFP-AHK5* construct were infiltrated into the abaxial side of the leaf and the GFP fluorescence analysed by CLSM 2 days later. Top panels  =  GFP images, lower panels  =  GFP image overlaying bright field image. The scale bar represents 20 μm. Images shown from repeat experiments. (C) *AHK5* gene structure, position of primers used for genomic and RT-PCR and T-DNA insertions in *ahk5-1* (Col-0) and *ahk5-3* (Ws4). (D) Analysis of *AHK5* expression in seedlings of wild type, *ahk5-1* and *ahk5-3* mutant, *P_35S_-AHK5* complemented and AHK5 over-expressing plants using the indicated primer pairs (see Supplementary data for sequences).

The expression profile of *AHK5* in different *Arabidopsis* tissues and cell types was analysed by semi-quantitative RT-PCR. *AHK5* transcript was detectable in light-grown but not in etiolated seedlings (Fig. 2A). Furthermore, *AHK5* transcript was present in flowers, siliques and roots and to a lower extent in stems and leaves of 30-days-old *Arabidopsis* plants (Fig. 2A). Increasing the number of PCR cycles showed a detectable level of *AHK5* transcript in mature leaves. As guard cells were the focus of our study, *AHK5* expression was also analysed in guard-cell enriched samples [16]. Compared to whole leaves the *AHK5* transcript level was significantly lower in guard cells. However, *AHK5* expression was increased in guard cell RNA extracted from H_2_O_2_-treated leaves (Fig. 2A), suggesting that AHK5 might have a function in H_2_O_2_ signalling in guard cells. The guard cell expression of AHK5 was confirmed by creating an *AHK5* promoter-GFP-AHK5 genomic construct (*P_AHK5_:GFP-AHK5*) and transiently expressing this in tobacco leaves. As shown in Fig. 2B and comparable to our RT-PCR results, GFP fluorescence was detected in guard cells as well as in epidermal cells indicating that the *AHK5* promoter is active in stomata. Our expression data therefore correlate well with the expression profile of *AHK5* observed in the AtGenExpress developmental data set [28] and adds to that reported earlier [24].

### Identification and characterisation of *ahk5* T-DNA insertion mutants

The functional characterisation of *AHK5* was initiated by the isolation and molecular characterization of *Arabidopsis ahk5* T-DNA insertion lines. Two independent *ahk5* alleles were found, one in the INRA-Versailles T-DNA collection (*ahk5-3* in Ws4 background; [29]) and one in the Syngenta SAIL T-DNA collection (*ahk5-1* in Col-0 background; [30]), respectively. Plants homozygous for the insertion events were identified by PCR on genomic DNA, and the positions of the T-DNA insertions confirmed by sequencing. As shown in Fig. 2C the T-DNA of *ahk5-3* is located in an intron within the predicted HK domain, whereas in *ahk5-1* the T-DNA is inserted in a 3′ exon which encodes a part of the AHK5 receiver domain. The T-DNA insertions did not appear to cause any other changes within the *AHK5* sequence. In addition, the presence of a single T-DNA insertion event in each line was verified by Southern blotting (Data S1). Semi-quantitative RT-PCR was used to determine the level of expression of *AHK5* in the homozygous lines. Fig. 2D shows that across the T-DNA borders there was no amplification of an *AHK5* transcript. Further extensive PCR using different primer pair combinations (of those shown in Fig. 2C) confirmed that no full-length transcript of *AHK5* was present in the mutant lines (Data S1). Therefore a fully functional AHK5 is unlikely to be expressed in *ahk5-1* and *ahk5-3*.

For complementation and functional analyses *ahk5-1* and *ahk5-3* were transformed with a construct expressing the full-length *GFP-AHK5* or *AHK5-TAP* fusion construct respectively under the control of the 35S promoter. In addition, *AHK5* was ectopically expressed in the Ws4 wild type background (Fig. 2D). RT-PCR confirmed that the transformed *ahk5* mutants and wild type expressed *AHK5* to high levels (Fig. 2D).

### A functional AHK5 HK is required for H_2_O_2_ responses in stomatal guard cells

**Figure 3:**
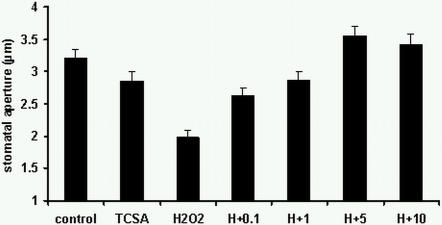
HK activity is required for H_2_O_2_-induced stomatal closure. Effect of the histidine kinase (HK) inhibitor 3,3′,4′,5-tetrachlorosalicylanilide on H_2_O_2_-induced stomatal closure in wild type *Arabidopsis* (Col-0). *Arabidopsis* leaves were incubated in stomatal opening buffer for 2.5 h followed by treatment for 15 min with 0.1, 1, 5 or 10 μM of TCSA prior to exposure to 200 μM H_2_O_2_ (H) for 2.5 h. Control, buffer alone. TCSA, buffer with TCSA alone at 10 μM. Data are expressed as mean +/− S.E. from 3 independent experiments (n = 60–80 guard cells).

Initially, a pharmacological approach was used to establish whether HK activity is required for H_2_O_2_ -induced stomatal closure. *Arabidopsis* wild type (Col-0) leaves were pre-treated with the inhibitor 3,3′,4′,5-tetrachlorosalicylanilide (TCSA) [31], followed by exposure to H_2_O_2_ and stomatal apertures measured. TCSA inhibited H_2_O_2_-induced closure in a dose-dependent manner (Fig. 3), thereby suggesting that HK activity is indeed required for H_2_O_2_ signalling to occur in guard cells leading to stomatal closure.

**Figure 4:**
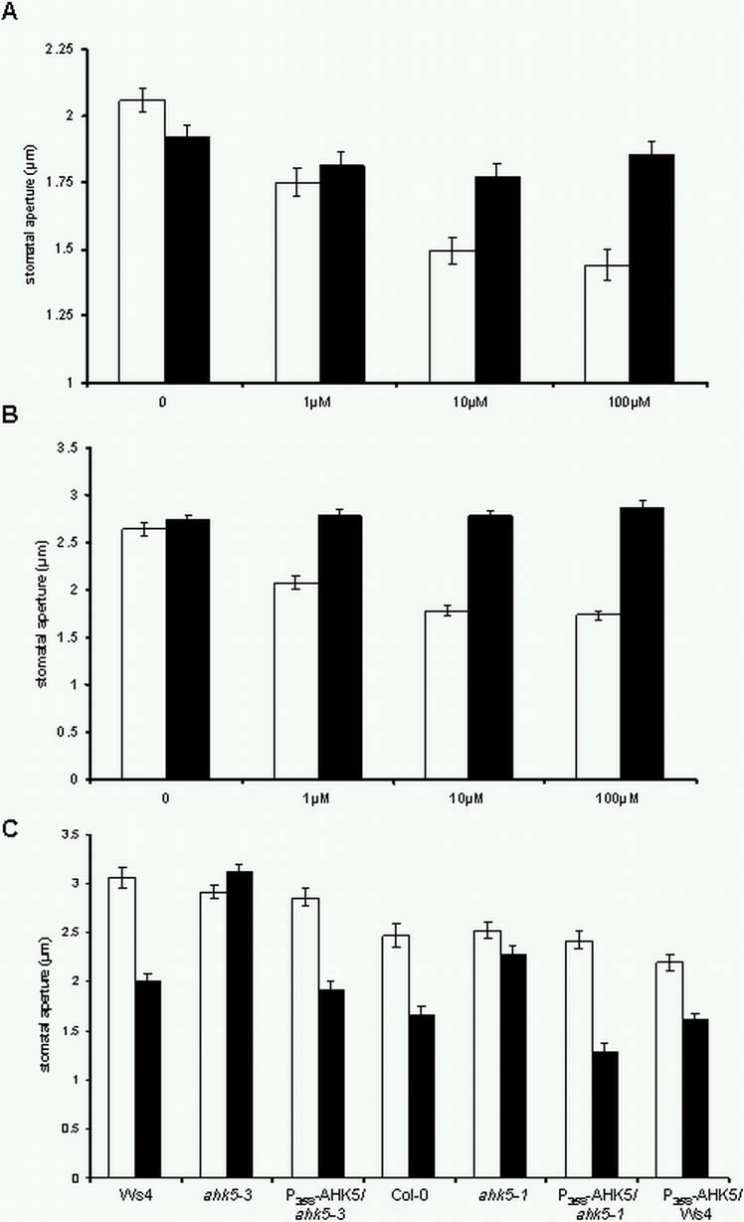
*ahk5* mutant guard cells show reduced sensitivity to H_2_O_2_. (A) Stomatal closure in wild type Col-0 (white bars) and *ahk5-1* (black bars) leaves 2.5 h after exposure to increasing concentrations of H_2_O_2_. (B) Stomatal closure in wild type Ws4 (white bars) and *ahk5-3* (black bars) leaves 2.5 h after exposure to increasing concentrations of H_2_O_2_. (C) Stomatal closure in leaves of wild type (Ws4, Col-0), *ahk5-3* and *ahk5-1* as well as in the *ahk5* mutants and wild type (Ws4) transformed with a construct expressing the *AHK5* cDNA under the control of the *35S* promoter (*P_35S_-AHK5*). The leaves were either mock-treated (white bars) or exposed to or 100 μM H_2_O_2_ (black bars). Data are expressed as mean +/− S.E. derived from measuring the apertures of at least 60 guard cells from 3 independent experiments.

Regulation of *AHK5* expression in guard cells by H_2_O_2_ led us to investigate whether AHK5 function is necessary for the response of guard cells to exogenous H_2_O_2_ by performing stomatal bioassays. Compared to wild type, guard cells of *ahk5-1* and *ahk5-3* were dramatically less sensitive to H_2_O_2_ at various concentrations (Fig. 4 A, B). The sensitivity of both mutant lines to H_2_O_2_ was restored by the expression of the wild type *AHK5* cDNA under the control of the 35S promoter (Fig. 4C) showing that the GFP-AHK5 fusion protein used for the localisation studies is functional *in planta*. These data also demonstrate that loss of *AHK5* gene function causes the H_2_O_2_-insensitive mutant phenotype and that AHK5 is required for stomatal response to exogenous H_2_O_2_. Interestingly, ectopic expression of *AHK5* in wild type background resulted in slightly smaller stomatal apertures compared to wild type Ws4 in the absence of H_2_O_2_ (Fig. 4C, right columns). This suggests that the guard cells of the *AHK5* overexpressor are more sensitive to endogenous H_2_O_2_. However, we observed a response of the overexpressor to exogenous H_2_O_2_ suggesting that ectopic accumulation of AHK5 *per se* does not significantly alter the plant's sensitivity to H_2_O_2_.

### Stomatal responses to NO, ethylene and darkness are impaired in ahk5 mutants

**Figure 5:**
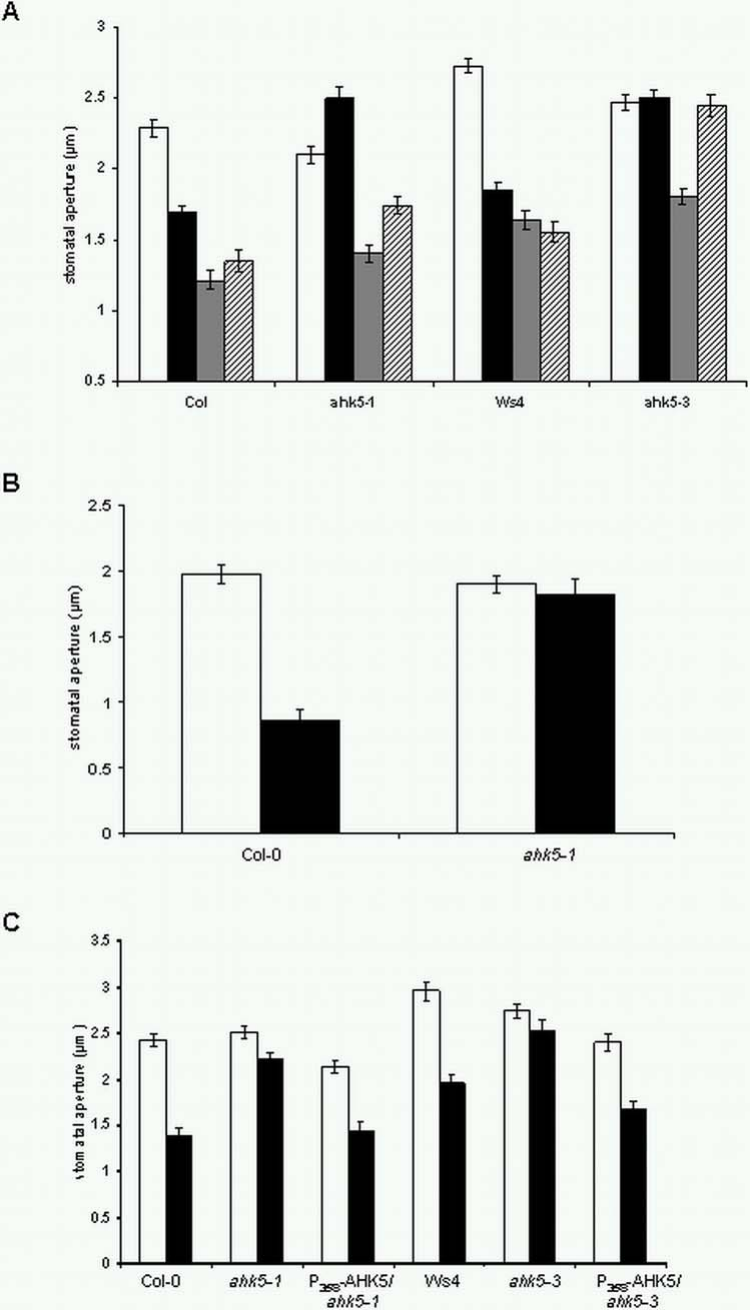
Guard cells of *ahk5* mutants show an altered response to NO, ethylene and darkness. (A) Stomatal closure in wild type (Col-0, Ws4), *ahk5-3* and *ahk5-1* in response to buffer (white bars), 50 μM sodium nitroprusside, (SNP, black bars), 10 μM abscisic acid (ABA, grey bars) and 2.5 h exposure to darkness (striped bars). Data from *ahk5-1* dark treatment are statistically significant (Student's t-test; p<0.05) versus dark treatment of wt, and data from *ahk5* ABA treatment are statistically significant (p<0.05) versus controls. (B) Guard cell response in plant-attached leaves in light and after transfer to darkness. Stomatal apertures were measured from wild type (Col-0) or *ahk5-1* leaves either 30 min prior to light off (white bars) or 1 h after transfer to darkness (black bars). Data are expressed as mean +/− S.E. from 3 independent experiments (n = 60 guard cells). (C) Stomatal closure response to ethylene in guard cells of wild type (Col-0, Ws4), *ahk5-1*, *ahk5-3* and *ahk5* mutants transformed with the *P_35S_-AHK5* construct. White bars, mock-treated; black bars, 3 h treatment with 100 μM ethephon. Data are expressed as mean +/− S.E. from 3 independent experiments (n = 60 guard cells).

The insensitivity of *ahk5* mutant guard cells to H_2_O_2_ suggests that AHK5 could be an essential signalling component in the stomatal closure response of *Arabidopsis* to various stimuli. Recently, we have shown that H_2_O_2_ induces the generation of NO in the response of guard cells to ABA [32]. If AHK5 is acting downstream of H_2_O_2_, the response to NO might also be affected in the *ahk5* mutants. As shown in Fig. 5A, *ahk5-1* and *ahk5-3* stomata showed a reduced sensitivity to the NO donor sodium nitroprusside (SNP). The NO insensitive phenotype of the *ahk5* mutants was functionally complemented by the wild type *GFP*-*AHK5* construct (Fig. S1). In addition, experiments with TCSA showed that NO-induced closure required HK activity (Fig. S2). These results indicate a function for AHK5 in both the H_2_O_2_ and NO response pathway in *Arabidopsis* guard cells. In contrast, the sensitivity of both mutants to ABA was not changed appreciably (Fig. 5A) suggesting the possibility that stimuli other than ABA lead to H_2_O_2_ and NO generation which might act via AHK5, and that ABA signalling occurs largely independent of AHK5.

In our previous work we demonstrated that pea guard cells exposed to darkness generate H_2_O_2_ [33]. Our pharmacological data revealed that pre-treatment with TCSA inhibited dark-induced stomatal closure, suggesting a requirement for HK activity in this response (Fig. S2). To investigate the role of AHK5 in dark-induced stomatal closure, the response of *ahk5* mutants to dark conditions were examined. As shown in Fig. 5A, detached leaves of both the *ahk5-1* and *ahk5-3* mutants showed reduced stomatal closure in response to dark conditions, with stomata of *ahk5-1* responding slightly to dark conditions. The experiments were also performed with non-detached leaves. Whilst the stomata of wild type and *ahk5-1* plants were open 30 min prior to transfer to darkness, the light-off conditions induced stomatal closure only in the wild type but not in the mutant (Fig. 5B). These data indicate that AHK5 function also contributes to the dark-induced stomatal closure response in *Arabidopsis*.

We have shown previously that ethylene perceived by the ethylene receptor ETR1 (a hybrid HK) also induces stomatal closure via H_2_O_2_ synthesis [16]. Furthermore, a functional ETR1 receptor is required to mediate H_2_O_2_-induced stomatal closure [15]. Experiments with TCSA showed that HK activity is required for ethylene-induced stomatal closure (Fig. S2). We therefore investigated the effect of ethylene on *ahk5-1* and *ahk5-3* guard cells. Treatment with the ethylene-generating compound ethephon induced a stomatal closure response in wild type but not in *ahk5* guard cells. The sensitivity to ethylene could be restored when the GFP- and TAP-tagged *AHK5* fusion constructs were expressed in the *ahk5* mutants under the control of the 35S promoter (Fig. 5C). These data show that ethylene induces stomatal closure via AHK5 function.

**Figure 6:**
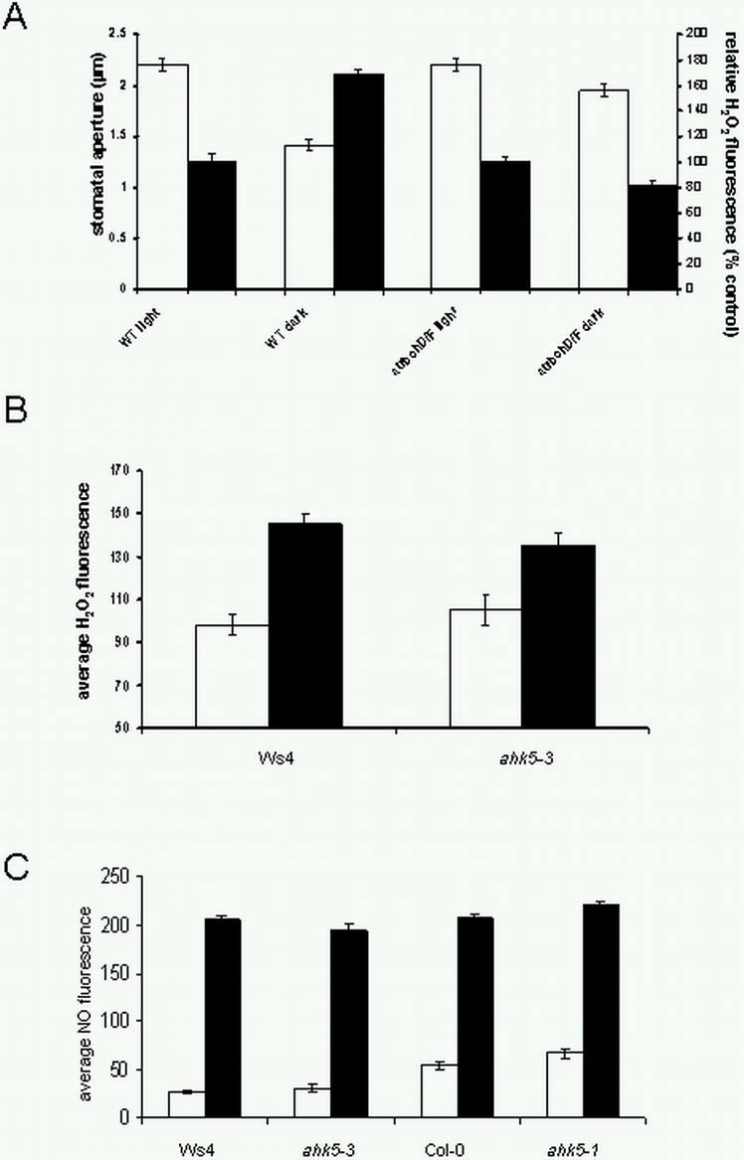
Regulation of H_2_O_2_ homeostasis by AHK5. (A) Stomatal responses to darkness in wild type (Col-0) and the *atrbohD/F* mutant. White bars, stomatal apertures of guard cells from leaves exposed to light (light) or transferred to darkness for 2 h (dark). Black bars, H_2_O_2_ fluorescence determined by confocal microscopy from epidermal peels exposed to light (light) or transferred to darkness for 30 min (dark). Data are expressed as mean +/− S.E. (n = 60 guard cells for aperture measurements; n = 40–80 guard cells for average H_2_O_2_ fluorescence from confocal experiments). (B) Darkness-induced H_2_O_2_ synthesis in wild type (Ws4) and *ahk5-3* mutant guard cells. H_2_O_2_ fluorescence from epidermal peels exposed to ambient light (white bars) or darkness (black bars) for 30 min was determined by confocal microscopy using the H_2_O_2_-sensitive fluorescent dye H_2_-DCFDA. Data are expressed as mean +/− S.E. (n = 40–80 guard cells for each treatment). (C) H_2_O_2_-induced NO fluorescence from guard cells of wild type and *ahk5* mutants using confocal microscopy and the NO-sensitive fluorescent dye DAF2-DA. White bars, mock-treated; black bars, 15 min treatment with 100 μM H_2_O_2_. Data are expressed as mean +/− S.E. (n = 75–130 guard cells for each treatment.

The data so far indicate that AHK5 function is required for H_2_O_2_, NO, darkness and ethylene-induced stomatal closure, but not for ABA-induced closure. To establish if AHK5 might be regulating redox homeostasis in response to these stimuli, the generation of both H_2_O_2_ and NO were measured in guard cells. Whilst the source of H_2_O_2_ in *Arabidopsis* guard cells has been established as *AtrbohD* and *F* for ABA [13], and *AtrbohF* for ethylene [16], it is not known how darkness induces H_2_O_2_. Figure 6A shows that guard cells of the *atrbohD/F* NADPH oxidase double mutant did not produce H_2_O_2_ nor close their stomata after transfer into darkness, thereby demonstrating that these homologs regulate dark-induced H_2_O_2_ synthesis in *Arabidopsis* guard cells. Interestingly, *ahk5* guard cells did generate H_2_O_2_ following dark adaptation of leaves as in wild type (Fig. 6B), thereby suggesting that AHK5, although involved in dark-induced stomatal closure, is not involved in regulating dark-mediated H_2_O_2_ synthesis.

**Figure 7:**
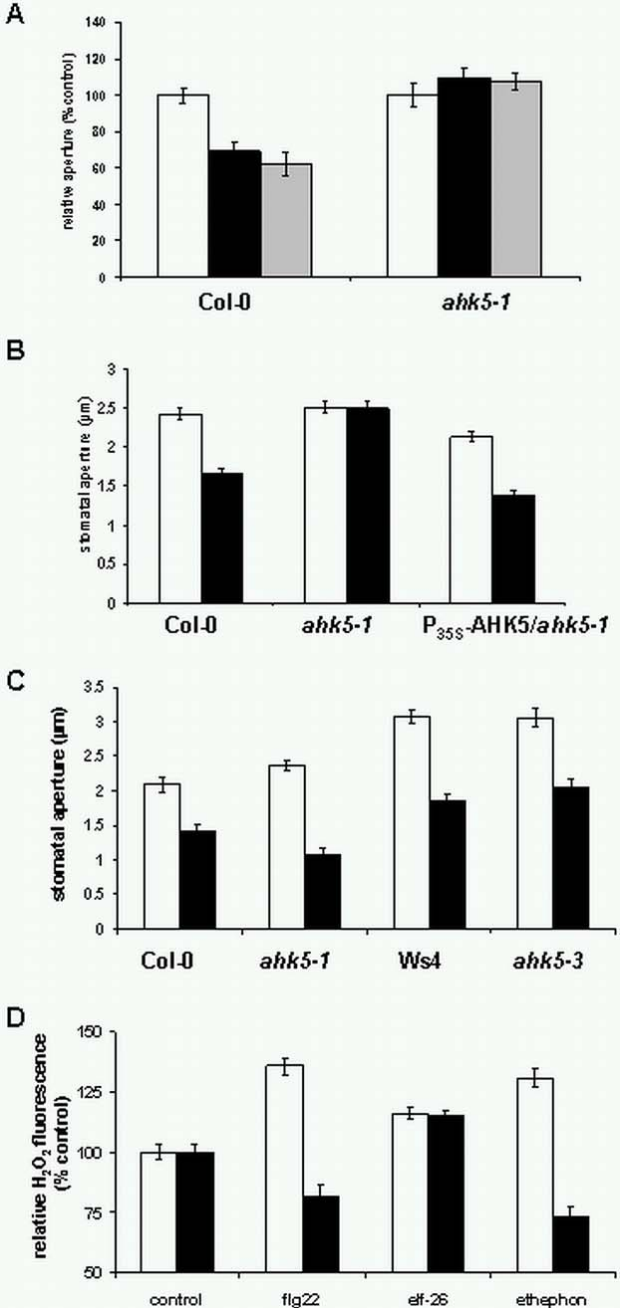
Guard cells of *ahk5* mutant show a differential response to PAMPs. (A) Stomatal aperture of wild type (Col-0) and *ahk5-1* 3 h after exposure to *P. syringae* pv. DC3000 (black bars) or hrpA*^−^* mutant (grey bars). White bars, mock-treatment. (MgCl_2_). (B) Stomatal closure of wild type (Col-0), *ahk5-1* and *ahk5-1* transformed with a *P_35S_-AHK5* construct after a 3 h treatment with either buffer (white bars) or 10 nM flg22 peptide (flg22, black bars). (C) Stomatal closure of wild type (Col-0, Ws4), *ahk5-1* and *ahk5-3* in response to a 3 h treatment with buffer (white bars) or 1 μM elf26 peptide (elf26, black bars). Data are expressed as mean +/− S.E. from 3 independent experiments (n = 60 guard cells), p<0.05 (student's t-test) versus appropriate controls. (D) H_2_O_2_ fluorescence from epidermal peels of wild type (Col-0, white bars) and *ahk5-1* (black bars) treated for 15 min with 10 nM flg22, 1 μM elf26 or for 30 min with 100 μM ethephon. Fluorescence intensity was quantified as described in methods. Data are expressed as relative fluorescence (% control values) +/− S.E. (n = 90–122 guard cells for each treatment).

H_2_O_2_ can also induce NO synthesis in the ABA signal transduction pathway in guard cells [32]. Although *ahk5* mutants do not respond to either H_2_O_2_ or NO (Fig. 4 and 5A), H_2_O_2_ induced NO synthesis in both mutant alleles (Fig. 6C), thereby positioning AHK5 downstream of H_2_O_2_ and NO in the signal response pathway. In addition, ethylene-induced H_2_O_2_ production was investigated in *ahk5-1*. Upon treatment with ethephon we observed a strong increase in H_2_O_2_-fluorescence in wild type guard cells (Fig. 7D). In contrast, ethylene caused a decrease of H_2_O_2_ levels in *ahk5-1* in comparison to that of the mock-treated control (Fig. 7D). These data demonstrate that AHK5 contributes to both, the ethylene-induced H_2_O_2_ production and stomatal closure response in *Arabidopsis*. However, ABA induced H_2_O_2_ synthesis in *ahk5* mutant, as in wild type guard cells (25% increase over controls; Data S1).

In summary so far, the data presented show that darkness-induced H_2_O_2_ synthesis, which is generated by ATRBOHD/F, signals through AHK5 to mediate stomatal closure, and that AHK5 is positioned downstream of H_2_O_2_ and NO in the signal response pathway. Moreover, AHK5 regulates ethylene-induced H_2_O_2_ synthesis leading to stomatal closure, but is not involved in the ABA signal transduction pathway.

### Stomatal responses to PAMPs are impaired in the ahk5 mutants

Bacteria-derived pathogen-associated molecular patterns (PAMPs) such as flagellin and EF-Tu have been shown to induce the synthesis of ethylene [34] and an oxidative burst in *Arabidopsis* leaves, which is mediated by ATRBOHD [35]. Furthermore, recent work by Melotto and colleagues (2006) demonstrated that stomata act as sites of entry for bacteria, and that bacteria and bacteria-derived PAMPs caused a primary stomatal closure response followed by a re-opening, when the plant was attacked by a virulent bacterium [36].

Experiments with the *Pseudomonas syringae* strains *pv* DC3000 and the *hrpA^−^* mutant, which lacks a functional type III secretory apparatus, revealed that *ahk5-1* mutant guard cells showed reduced stomatal closure in response to both bacterial strains (Fig. 7A). The reduced stomatal closure response to the *hrpA^−^* mutant strain suggests that AHK5 is involved in the basal defence of *Arabidopsis*, which is mediated by PAMPs such as the flagellin peptide 22 (flg22) and the EF-Tu peptide 26 (elf26; [37]).

Flagellin-induced stomatal closure in wild type plants was inhibited by pre-treatment with TCSA (Fig. S2), indicating a requirement for HK activity to mediate this response. To investigate this in more detail, leaves of the *ahk5-1* mutant (Col-0 background) were exposed to flg22 and the stomatal apertures measured. The response to flg22 was not investigated in the *ahk5-3* mutant as the Ws background lacks a functional FLS2 receptor [38]. As reported [36], flg22 induced stomatal closure in Col-0 wild type guard cells (Fig. 7B). This response was not observed in the *ahk5-1* mutant. The loss-of-function phenotype of *ahk5-1* was complemented by the expression of the *GFP-AHK5* fusion construct, which is under the control of the 35S promoter (Fig. 7B). Interestingly, elf26 caused an identical stomatal closure response in wild type (Col-0, Ws4) as well as in the *ahk5-1* and *ahk5-3* mutants (Fig. 7C). These data suggest that AHK5 plays a specific role in the flagellin signal response pathway in guard cells.

PAMP-induced H_2_O_2_ production was also investigated to study the role of AHK5 in redox homeostasis during basal defence. Interestingly, whereas elf26 caused an identical increase in H_2_O_2_ fluorescence in guard cells of wild type and *ahk5-1*, treatment with flg22 actually caused a decrease in H_2_O_2_ fluorescence in *ahk5-1*, when compared with that of the mock-treated control (Fig. 7D). Therefore AHK5 appears to play a role in both the flg22-induced regulation of H_2_O_2_ production and stomatal closure.

## Discussion

### An AHK5-dependent signalling pathway acts in stomatal guard cells

We have characterised the canonical HK AHK5 as being a cytoplasmic/membrane protein differentially expressed in various tissues of *Arabidopsis*. Despite the predicted cytoplasmic location [18] and lack of transmembrane domains within the AHK5 sequence, our data suggest that AHK5 co-localises at the plasmalemma as well. Interestingly, putative N-myristoylation sites are predicted for AHK5, suggesting that an association of AHK5 to the plasmalemma is possible. In addition, we cannot exclude the possibility that the interaction with intrinsic membrane proteins including other HKs locates AHK5 to the plasmalemma.

Expression of *AHK5* appears to be regulated by H_2_O_2_ in guard cells. In addition, we have shown using functional approaches that AHK5 plays a crucial role in mediating H_2_O_2_-dependent processes in stomatal guard cells which are induced by environmental and hormonal signals such as NO, ethylene, adaptation to darkness and the PAMP flagellin (flg22). Despite its low level of expression, mutations in the *AHK5* gene appear to have profound effects on the guard cell phenotype. The pattern of low expression of a gene in guard cells still resulting in distinct stomatal phenotypes has been observed before – e.g. the nitric oxide (NO)-generating enzyme nitrate reductase (NR). There are two *NR* genes, *NR1* and *NR2*, in the *Arabidopsis* genome. Although *NR1* is expressed at much lower levels than *NR2* in guard cells, mutations in NR1 but not NR2 appear to affect ABA-induced NO responses in stomata [39]. Although *cis* elements found in the promoters of guard cell specific genes [40] are present in the promoter region of *AHK5*, it is possible that other *cis* elements which repress promoter activity in guard cells cause the weak expression pattern, as seen with other guard cell genes [41]. It is also likely that phenotypic effects may be explained by specific protein-protein interactions between a low-expressed protein in guard cells and other proteins of higher abundance. Preliminary data show that expression of the phosphotransfer protein AHP2 and the response regulator ARR4 that AHK5 interact with are of higher abundance than *AHK5* in guard cells (Data S1). Further studies will analyse the functions of *AHK5* promoter, as well as studying the protein interacting two-component partners of AHK5.

### AHK5 functions as integrator of H_2_O_2_- dependent signalling in stomatal guard cells

A lack of functional AHK5 results in altered stomatal responses not only to exogenous H_2_O_2_ but also to multiple stimuli which are known to generate H_2_O_2_ in plant tissues. These signals include ethylene [16], the light-off signal (darkness; data shown here and [33]) and the PAMP flg22 [35].

Previously we provided evidence for a role for the plant hormone ethylene in mediating stomatal closure *via* H_2_O_2_ signalling [16]. Although the HK function of ETR1 is not required for H_2_O_2_ signalling, the N-terminus of ETR1 appears to be essential for this signalling to occur in guard cells [15]. The pharmacological data presented here with TCSA indicate that HK activity is required for H_2_O_2_ (and NO)-induced stomatal closure in *Arabidopsis*. As ethylene is able to produce H_2_O_2_ in wild type guard cells [16], and as shown here, AHK5 is also involved in ethylene-dependent signalling leading to H_2_O_2_ synthesis and stomatal closure, it is possible that the ethylene-sensing N-terminus of ETR1 functionally and/or physically interacts with H_2_O_2_-activated AHK5 during ethylene signal transduction in guard cells. This is in agreement with recent work by Iwama et al. [24], who demonstrated a functional interaction of *AHK5* with the ethylene and ABA response in the control of root growth in *Arabidopsis*. The authors propose an “unidentified” stimulus as being sensed by AHK5, which could integrate the ABA and ethylene signalling pathways in roots. On the basis of our data it is likely that this unknown stimulus for AHK5 is H_2_O_2_, although the *ahk5* phenotype in roots in response to H_2_O_2_ remains to be determined.

Synthesis of H_2_O_2_ upon transfer of plants to darkness was found to depend on NADPH oxidase orthologues in pea [33]. By using an *atrbohD/F* double mutant we demonstrate here that dark-induced H_2_O_2_ formation occurs by a similar mechanism in *Arabidopsis*. We also show that *ahk5* mutants do not close their stomata in response to darkness. This is substantiated by the pharmacological data showing inhibition by TCSA, of dark-induced closure in wild type *Arabidopsis*. Stomata of the H_2_O_2_-insensitive *etr1-1* mutant still respond to darkness (Data S1), suggesting the possibility that as far as HKs are concerned, AHK5 might have a unique role in the dark-H_2_O_2_ signalling pathway in guard cells. Although of fundamental physiological and ecological relevance, little is known of the dark-induced signalling processes leading to stomatal closure. The type 2C protein phosphatases ABI1 and ABI2 [42], the outward potassium channel GORK [43] and the MYB transcription factor AtMYB61 [44] have functions in guard cell responses in the dark. However, the mechanism by which AHK5-dependent phosphorelay is linked to proteins such as ABI1, 2, GORK and AtMYB61 in the guard cell signalling network is not yet known.

AHK5 also appears to be essential for mediating flagellin- (flg22) induced stomatal closure in the Col-0 ecotype, again correlating with the TCSA data demonstrating inhibition of flg22-induced stomatal closure. Surprisingly, the AHK5-mediated response seems to be specific for flg22 because the mutants showed a wild type stomatal closure response to the PAMP elf26. This is unexpected because the signalling cascades of flg22 and elf26 overlap considerably in non-guard cell tissue in respect to ethylene and H_2_O_2_ production, alkalinisation, activation of MAPKs and changes in gene expression [34], [37]. However, as noted recently, there are likely to be differences between guard cells and mesophyll cells mediating pre- and post-invasive immunity [45]. Recent work by Melotto et al. [36] indicates that bacterial PAMPs such as flagellin induce stomatal closure in *Arabidopsis*. Our data, therefore, position AHK5 in a signal transduction cascade specific to flagellin in guard cells. The exact mechanism by which AHK5 interacts with this pathway remains to be determined, but it is likely that the interaction occurs with the flg22 receptor FLS2. It is interesting to note that the expression of FLS2 is abundant in guard cells [46]. Given that FLS2 is a plasmalemma-bound and AHK5 is likely to be located at the plasma membrane as well as the cytoplasm, one may speculate that both receptors could physically interact at the plasma membrane allowing AHK5 to perceive high local H_2_O_2_ concentrations induced by the flg22-activated FLS2/BAK1 receptor complex [47], [48]. Importantly, flg22 but not elf26 was unable to induce H_2_O_2_ accumulation in *ahk5* mutant guard cells. Thus, AHK5 also appears to contribute to the flagellin-induced regulation of H_2_O_2_ levels in guard cells (see below for detailed discussion). This also implies that an additional H_2_O_2_ sensor is required for the perception of the H_2_O_2_ signal derived from the EF-Tu/EFR receptor complex.

As demonstrated by the wild type behaviour of both *ahk5* mutants to ABA, the AHK5-dependent signalling pathway does not contribute to the ABA response pathway in guard cells. This is not entirely surprising, as we have previously observed that mutants of ETR1 also respond normally to ABA [16]. Although ABA signalling requires the synthesis and action of H_2_O_2_ [13], our data indicate that this function is independent of AHK5.

### The complexity of redox signalling in stomatal guard cells

Redox signalling in guard cells is likely to be regulated *via* a number of signalling pathways. This may be because of the nature of H_2_O_2_ as such, being able to diffuse freely between cellular compartments, and also due to the fact that H_2_O_2_ is likely to be generated in localised “hot spots” within the cell, thereby leading to localised effects of H_2_O_2_ on its target proteins [2]. Generation of H_2_O_2_ in guard cells in response to ABA and darkness occurs *via* the NADPH oxidase orthologues ATRBOHD and ATRBOHF ([13] and this study). ATRBOHF is essential for H_2_O_2_ generation in response to ethylene and ETR1 functions as a central mediator of H_2_O_2_ responses [16]. In leaves, flg22-induced H_2_O_2_ production occurs *via* ATRBOHD [35]. RBOH proteins are localised at the plasma membrane [49], and the proteins involved in H_2_O_2_ signalling are located at the ER (ETR1; [25], [50]), plasma membrane (AHK5, FLS2, [51]) or in the cytosol (AHK5). We have observed that AHK5 function is crucial for ethylene and flg22-induced but not for darkness or elf26-induced H_2_O_2_ accumulation. Further work using *ahk5* plants crossed with *atrboh* mutants is required to confirm how AHK5 and RBOH signalling interact. However, *AHK5* transcript appears to be regulated by H_2_O_2_ in guard cells, and AHK5 function is essential for H_2_O_2_ and NO-dependent signal transduction. Together, the data suggest that AHK5 acts to maintain H_2_O_2_/redox homeostasis in guard cells in response to multiple stimuli. A positive feedback loop is possible, whereby H_2_O_2_, generated *via* different stimuli (from RBOH), regulates AHK5 expression (or activity), which in turn regulates H_2_O_2_ synthesis (for ethylene and flagellin pathways) and action leading to stomatal closure. Regulation of the expression of HKs by the stimulus that induces their activity is not uncommon – expression of the cytokinin receptor CRE1 and ethylene receptors is regulated by cytokinin and ethylene treatments, respectively [22]. Detailed investigations are necessary to elucidate the molecular mechanisms of redox regulation of AHK5, *via* mass spectrometry of the purified protein.

The dual function of AHK5, in regulating H_2_O_2_ synthesis and action is reminiscent of the role of ETR1, which we have shown previously to have a dual function in guard cells, that of perceiving ethylene as well as H_2_O_2_ [16]. AHK5 could therefore have multiple functions as well: firstly, AHK5 may contribute to the flagellin- and ethylene-induced H_2_O_2_ accumulation and, secondly, may sense H_2_O_2_ produced in the course of the ethylene-, NO-, flg22- and darkness-regulated stomatal closure response. Our evidence that AHK5 plays a role in the inducible accumulation of H_2_O_2_ comes from our observation that the H_2_O_2_ level is decreased in *ahk5-1* guard cells upon treatment with ethylene or flg22. It is not yet known whether AHK5 interacts with receptors such as ETR1 or FLS2, H_2_O_2_-generating enzymes such as ATRBOHD/F and redox-active proteins such as ATGPX3, ABI1 and ABI2 which are located in distinct sub-cellular compartments [9], [10], [52] or whether AHK5 co-ordinates their functions to integrate H_2_O_2_ signalling. However, our study provides evidence that AHK5 acts to integrate multiple H_2_O_2_-dependent processes at different molecular levels.

### Summary and concluding remarks

We have shown that AHK5 functions in guard cells to mediate stomatal responses to various stimuli that generate H_2_O_2_. Evidence is slowly emerging that implicate overlapping signalling pathways during abiotic and biotic stress responses in plants, which include hormone signalling, ROS signalling and protein phosphorylation [22], [53]. Our data position AHK5 both upstream and downstream of ROS in integrating bacterial, darkness and hormonal-induced responses which could be achieved by differential protein-protein interactions. This is the first demonstration of a role for a HK two-component signalling pathway in integrating abiotic and biotic signals. Moreover, it is the first identification of a HK mediating H_2_O_2_ homeostasis to integrate multiple stress responses in guard cells. The data presented here highlight the mechanism and function of the AHK5 two-component signal transduction pathway in stomata, which are ideal model systems to study integration of multiple stimuli.

## Materials and Methods

### Growth and maintenance of plants

Wild type and mutant seeds of *Arabidopsis thaliana* ecotype Columbia (Col-0) and Wassilewskijia (Ws4) were sown on Levington's F2 compost and grown under a 16 h photoperiod (100–150 μE m*^−^* ^2^ s*^−^* ^1^), 22°C and 65% relative humidity in controlled environment growth chambers (Sanyo Gallenkamp, UK). *atrbohD/F* seeds were obtained from J Jones (Sainsbury Laboratory, Norwich, UK).]. Details of the T-DNA insertion lines in *AHK5* are as follows: *ahk5-1* mutant seeds (SAIL 50_H11) were originally obtained from Syngenta (SAIL collection, now available at ABRC/NASC), and the *ahk5-3* seeds (FLAG_271G11) were obtained from the INRA/FLAG-FST collection at Versailles [29], [30].

### Stomatal bioassays

Stomatal assays were performed on leaves essentially as described in [15]. Leaves were floated for 2.5 h under continuous illumination (100–150 μE m*^−^* ^2^ s*^−^* ^1^) in Mes/KCl buffer (5 mM KCl/10 mM Mes/50 μM CaCl_2_, pH 6.15). Once the stomata were fully open, leaves were treated with various compounds for a further 2.5 h. The leaves were subsequently homogenised individually in a Waring blender for 30 s and the epidermal fragments collected on a 100 μm nylon mesh (SpectraMesh, BDH-Merck, UK). Stomatal apertures from epidermal fragments were then measured using a calibrated light microscope attached to an imaging system (Leica QWin software, Leica, UK). Flg22 and elf-26 peptides were a kind gift from J Mansfield (Imperial College London). Elicitors were added to the incubation buffer at 2.5 h and stomatal apertures measured after a further 3 h.

For bacterial experiments, *Pseudomonas syringae* pv DC3000 or *P. syringae* hrpA*^−^* mutant were grown overnight in LB media and overnight cultures centrifuged, resuspended in 10 mM MgCl_2_ at an OD_600_ = 0.2 (equivalent to 2×10^8^ cfu/ml). Silwet (0.002% v/v) was added to cultures or MgCl_2_ to act as a wetting agent. Bacteria were gently coated onto the abaxial side of leaves on intact plants (controls were MgCl_2_ with Silwet alone). Plants were left in the growth chambers with a covered lid (to increase humidity) for 3 h, inoculated leaves subsequently detached and stomatal apertures measured.

### Measurement of H_2_O_2_ and NO using confocal/fluorescent microscopy

Epidermal peels from mature leaves, prepared as described above, were incubated in Mes/KCl buffer for 2–3 h. Following this, the fragments were loaded by incubation in 50 μM of the H_2_O_2_-sensitive fluorescent dye 2′,7′-dichlorodihydrofluorescein diacetate (H_2_DCFDA, Molecular Probes, Leiden, The Netherlands) for 10 min. After washing in fresh buffer for a further 20 min, the fragments were challenged with various compounds as indicated in the figure legends. For dark treatments the peels were incubated in darkness for 30 min and microscopy performed. Confocal laser scanning microscopy was used to visualise fluorescence, using an excitation wavelength of 488 nm and an emission wavelength of 515–560 nm (Nikon PCM2000, Nikon Europe B.V. Badhoewvedorp, The Netherlands). Images were acquired and analysed using Scion Image software (Scion Corp., USA) to measure the relative fluorescence intensities in the cells following various treatments. For the data in Figure 7D, fluorescent microscopy (Zeiss Axioskop2, Zeiss, UK) was used with filter set 10 (excitation filter BP 450–490 nm, beam splitter FT 510 nm and emission filter BP 515–565 nm). Images were acquired and analysed using Image J software (NIH, USA). Data represent fluorescence intensities expressed as average fluorescence or as a percent of the control values, from several guard cells analysed in different experiments. For NO fluorescence, epidermal peels were loaded with 10 μM of the NO-sensitive dye diaminofluorescein diacetate (DAF2-DA, Calbiochem, UK) using exactly the same dye loading procedure, and images acquired using confocal microscopy as described above.

### Cloning, expression analysis of *AHK5* and characterisation of T-DNA mutants

The *AHK5* Entry clone was constructed using Gateway™ technology (Invitrogen, UK). It was obtained through TOPO-reaction using the pENTR/D-TOPO vector (Invitrogen). PCR was performed using Phusion™ polymerase (Finnzymes, UK) and cDNA from *Arabidopsis* roots as template. Primers were as follow: 5′-*CACC*-ATGGAGACTGATCAGATTGAGGAA-3′, 5′-GTGCAAATACTGTTGCAAACACTCTC-3′. The *AHK5* Entry clone was verified *via* restriction analysis and sequencing (GATC Biotech). The construct for the expression of the GFP fusion proteins under the control of 35S promoter (*P_35S_::GFP:AHK5*) was cloned *via* LR-reaction into the destination vector pK7WGF2.0 [54]. For complementation of the mutant line in the Ws4 background, the TAP-tag destination vector pYL436 [55] was used to transform *ahk5-3* plants. The transformants were selected on BASTA and gentamycin and analysed by PCR. For the Col-0 mutant complementation the *P_35S_::GFP-AHK5* construct in pK7WGF2.0 was used to transform *ahk5-1* plants. The transformants were selected on BASTA and kanamycin and analysed by PCR as described below. For the AHK5 overexpressor in Ws4 background, LR reaction was used to clone the *AHK5* cDNA under the control of 35S promoter into the destination vector pMDC32 [56]. All plant transformations were carried out using *Agrobacterium*-mediated transformation by floral dipping [57].

A four-step procedure was used to generate the *P_AHK5_::GFP::AHK5* expression cassette. In step 1, a 7117 bp fragment containing the 3.2 kb upstream promoter region of *AHK5*, the full length genomic sequence of *AHK5* and 217 bp of 3′ region was amplified from Col-0 genomic DNA by PCR using KOD Hot Start DNA polymerase (Novagen, Germany) and the primers *AHK.FOR.-3205* (5′-CACC-TCTAGACCCTACACGGGATAGATTATCG-3′) and *AHK.REV.+219* (5′-TTTGTCGACTCTGCTGGATTCGAATGGTGGG-3′) and cloned into the pENTRD/TOPO entry vector (Invitrogen, UK) to generate pMKC101. The entire construct was verified by sequencing. In step 2, a hybrid DNA fragment containing 643 bp upstream promoter region of *AHK5* from pMKC101 was joined to the *GFP* sequence and *AHK5* exon 1 sequence (from the *P_35S_::GFP::AHK5* construct) by single joint PCR [58]. Briefly, in the 1st round PCR stage, 2 separate PCR reactions were set up. In reaction 1, primer 1 (5′-CCTTTTGCATCTCGAGACTTCATGATTAC-3′) and primer 2 (5′-GGTGAACAGCTCCTCGCCCTTGCTCACCATTTCACAGACCATTGATCAAGGTTTCTC-3′) were used to incorporate a XhoI restriction site (underlined in primer 1) and a 27 bp of the 5′ end of *GFP* sequence (underlined in primer 2) onto the 5′- and 3′-ends of the 643 bp *AHK5* promoter region, respectively. In reaction 2, a fragment containing the entire *GFP* sequence and *AHK5* exon 1 was amplified using primer 3 (5′-ATGGTGAGCAAGGGCGAGGAGCTGTTCACC-3′) and primer 4 (5′-GATGAGTCGAATTCAATAGGTTTGGTAACC-3′) from the *P_35S_::GFP::AHK5* construct. Primer 4 contains an EcoRI site (underlined). The products from the two reactions were joined (*via* the 27 bp overlapping 5′ *GFP* sequence common to both PCR products) in the 2nd round PCR stage to generate a hybrid DNA fragment. This fragment was then amplified with the primers 1 and 4 in the 3rd round PCR stage. In step three, the hybrid PCR product was digested with XhoI/EcoRI, and cloned into an XhoI/EcoRI cut pMKC101 plasmid to create the *P_AHK5_::GFP::AHK5* cassette. The integrity of the hybrid DNA fragment was verified by sequencing. Finally, step 4; the *P_AHK5_::GFP::AHK5* cassette was cloned into the Gateway destination vector pMDC99 [56] using the LR reaction. This binary vector was then transformed into the *Agrobacterium tumefaciens* strain GV3101, and used in tobacco transient expression studies as described above.

For identification and characterisation of homozygous insertion mutants, genomic DNA isolated from appropriate wild type and mutant plants was used for PCR analysis, using various PCR combinations and primers. T-DNA primers used were those already described [29], [30]. Individuals were chosen from homozygote lines by selection on BASTA, Southern analysis confirmed the presence of single T-DNA insertions in these lines and at least 3 generations were followed through to get a homozygote population.

For RT-PCR, total RNA from corresponding tissues and developmental stages of *A. thaliana* was isolated using RNAwiz™ (Ambion, UK) or TRIZOL reagent (Invitrogen, UK) and genomic DNA was removed using TURBO DNA-free™ (Ambion, UK). RNA was isolated from guard cell-enriched epidermal fragments and whole leaves as described previously [16]. Subsequently, 1.5 μg of total RNA was reverse transcribed using oligo-dT primer with SuperScript™ III Reverse Transcriptase (Invitrogen, UK) and the resulting cDNA was used as template for the PCR with HotStart Taq polymerase (Genaxxon, Germany). PCR products were separated *via* agarose gel electrophoresis after different number of PCR cycles for comparison with *ACTIN2* or *EF1* as described in [59]. The sequences of the primers shown in Figure 2 are in Supplementary data.

### Transient transformation of tobacco leaf cells and *Arabidopsis* protoplasts, GFP and RFP analyses

The p19 protein from tomato bushy stunt virus cloned in pBIN61 [60] was used to suppress gene silencing in tobacco (*Nicotiana benthamiana)*. All plasmids were transformed in *Agrobacterium tumefaciens* strain GV3101 pMP90, which was grown in YEB medium to OD_600_ 1.0 and prior to infiltration resuspended in AS medium (10 mM MgCl_2_, 150 μM acetosyringone and 10 mM MES pH 5.7) to OD_600_ = 0.8. The *Agrobacterium* strains containing the GFP or p19 construct were mixed in a 1∶1 relationship and co-infiltrated into leaves of 4-week-old tobacco plants as described in [60]. The abaxial epidermis of infiltrated tobacco leaves was assayed for fluorescence by CLSM (confocal laser-scanning microscopy) 2 to 3 days post infiltration according to [61]. *Arabidopsis* protoplasts were transformed using PEG mediated transformation procedure and assayed for fluorescence by CLSM after 20 h [61]. CLSM was performed using a Leica TCS SP2 confocal microscope (Leica Microsystems, Germany). These CLSM images were obtained using the Leica Confocal Software and the HCX PL APO 63×/1.2 W CORR water-immersion objective.

For recording the RFP and GFP intensity profiles a homemade confocal laser-scanning microscope, based on a Zeiss Axiovert was used [62], [63]. The microscope was equipped with an avalanche photodiode (APD, SPCM-AQR-14, Perkin Elmer, USA) as a spectrally integrating detector. A pulsed 473 nm diode laser (Picoquant LDH-P-C470) operating at a repetition rate of 10 MHz served as excitation source. Fluorescence intensity images were obtained by raster scanning the sample and detecting emission intensity for every spot on the sampled area. The setup was equipped with a 480 nm long pass filter (Semrock Razor Edge LP02-473RU-25) to block back-scattered excitation light, a 500 nm bandpass filter (Semrock BrightLine BL500/24) to detect GFP-fluorescence and a 590 nm bandpass filter (Semrock FF01-590/20-25) to detect RFP-fluorescence in front of the APD. The processing of the obtained fluorescence intensity images was accomplished with the WSxM software [64].

### Protein extraction, cell fractionation, SDS-PAGE and western blotting

For cell fractionation 100 mg tissue of transiently transformed tobacco leafs were homogenised in liquid nitrogen and the homogenate was extracted in 2 ml homogenization buffer (25 mM MOPS, 0.1 mM MgCl_2_, 8 mM L-cysteine, 2.5 mM EDTA, 2× protease inhibitor mix (Roche), 250 mM sucrose; pH 7.8). The crude extract was cleared from debris by centrifugation (4000x*g*, 40 min, 4°C). The microsomal fraction was separated from the soluble fraction by ultracentrifugation (100,000x*g*, 30 min, 4°C). The pellet was washed three times in homogenization buffer supplemented with 0.05% Triton X-100 and resuspended in 50 μl SDS-PAGE sample buffer. The soluble fraction was mixed with SDS-PAGE sample (ratio: 2∶1 v/v). For SDS-PAGE 18 μl of the soluble fraction and 10 μl of microsomal fraction were loaded. Western blot analysis and immunodetection were performed according to [61] using anti-GFP antibody (Roche, Switzerland) to detect GFP-AHK5, BRI1-GFP, ERS1-GFP and ARR4-GFP. An anti-mouse-AP conjugate (BioRad, UK) was used as secondary antibody.

### Statistical analysis

All data from stomatal bioassays and fluorescence measurements were statistically analysed by using Student's t-test analysis. Data are statistically significant (p<0.01) for all treated versus control responses for wild type and complemented lines, and not significant (p>0.01) for mutant treated (H_2_O_2_, ethephon, NO, darkness and flg22) versus mutant controls, unless otherwise indicated.

## Supporting Information

Figure S1 The NO insensitive stomatal closure response phenotype of the ahk5-1 mutant is complemented by the 35S promoter-driven expression of the AHK5 cDNA. Stomatal closure in wild type Col-0, ahk5-1 mutant or ahk5-1 transformed with a construct expressing GFP-AHK5 under the control of the 35S promoter (P35S-AHK5/ahk5-1) in response to mock treatment (white bars) or SNP (50 μM, black bars) for 2.5 h.
(1.42 MB TIF)

Figure S2 Histidine kinase (HK) activity is required for NO-, dark- and flg22-induced stomatal closure. Effect of the HK inhibitor 3,3′,4′,5-tetrachlorosalicylanilide (TCSA) on stomatal closure in wild type Arabidopsis (Col-0). Arabidopsis leaves were incubated in stomatal opening buffer for 2.5 h followed by treatment for 15 min with 10 μM of TCSA prior to exposure to darkness, ethephon (eth, 100 μM), flg22 (100nM) or SNP (50 μM) for 2.5 h. Control, buffer alone. Data are expressed as mean +/− S.E. from 3 independent experiments (n = 60 guard cells).
(0.61 MB TIF)

Data S1 (0.02 MB DOC)
