# Supplementary figures and images for: The Histidine Kinase AHK5 Integrates Endogenous and Environmental Signals in Arabidopsis Guard Cells
Source: PLoS One. 2008 Jun 18;3(6):e2491. doi: 10.1371/journal.pone.0002491 (PMC2424244; doi:10.1371/journal.pone.0002491)

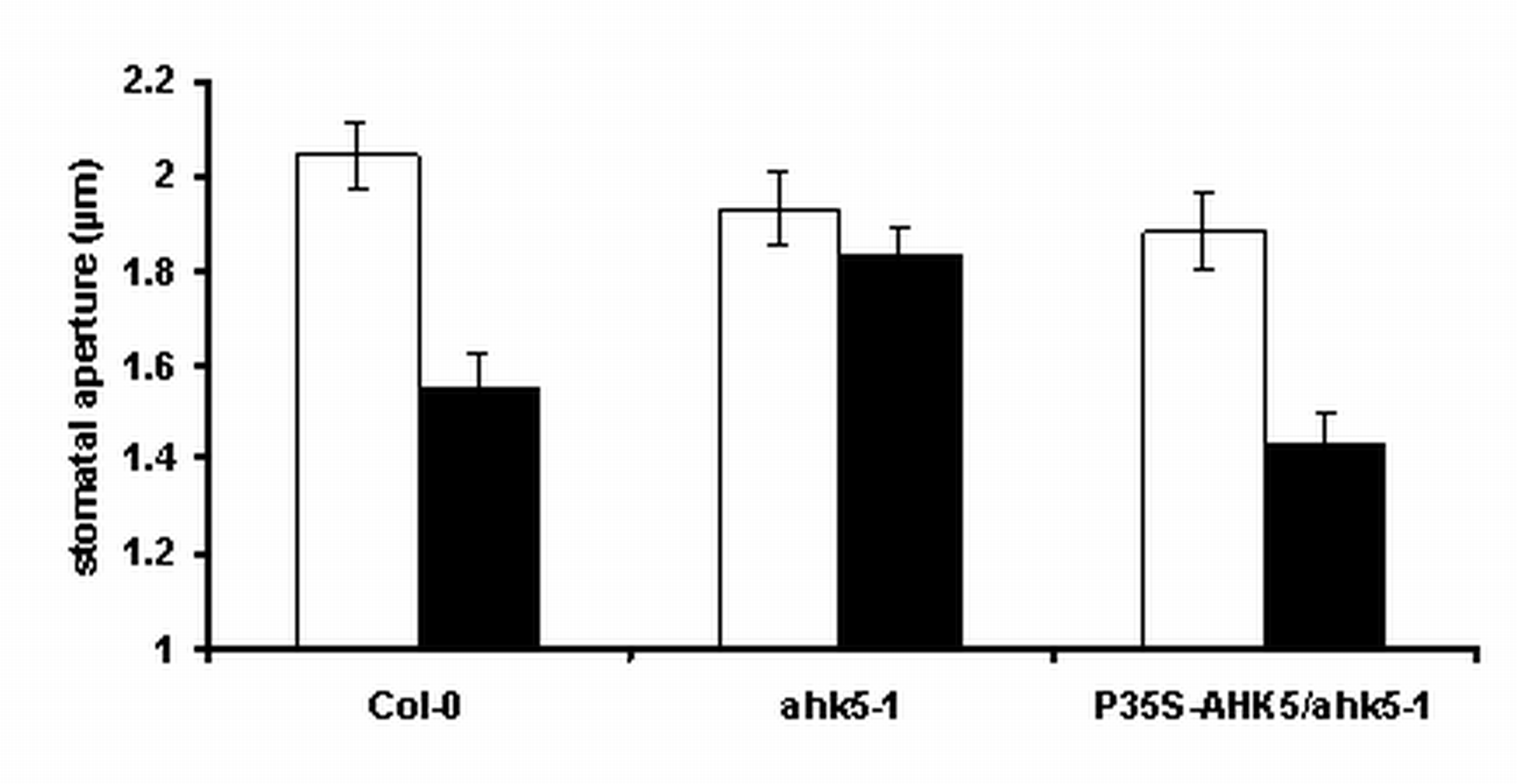

Supplement: Figure S1 — The NO insensitive stomatal closure response phenotype of the ahk5-1 mutant is complemented by the 35S promoter-driven expression of the AHK5 cDNA. Stomatal closure in wild type Col-0, ahk5-1 mutant or ahk5-1 transformed with a construct expressing GFP-AHK5 under the control of the 35S promoter (P35S-AHK5/ahk5-1) in response to mock treatment (white bars) or SNP (50 μM, black bars) for 2.5 h. (1.42 MB TIF) [file pone.0002491.s001.tif]

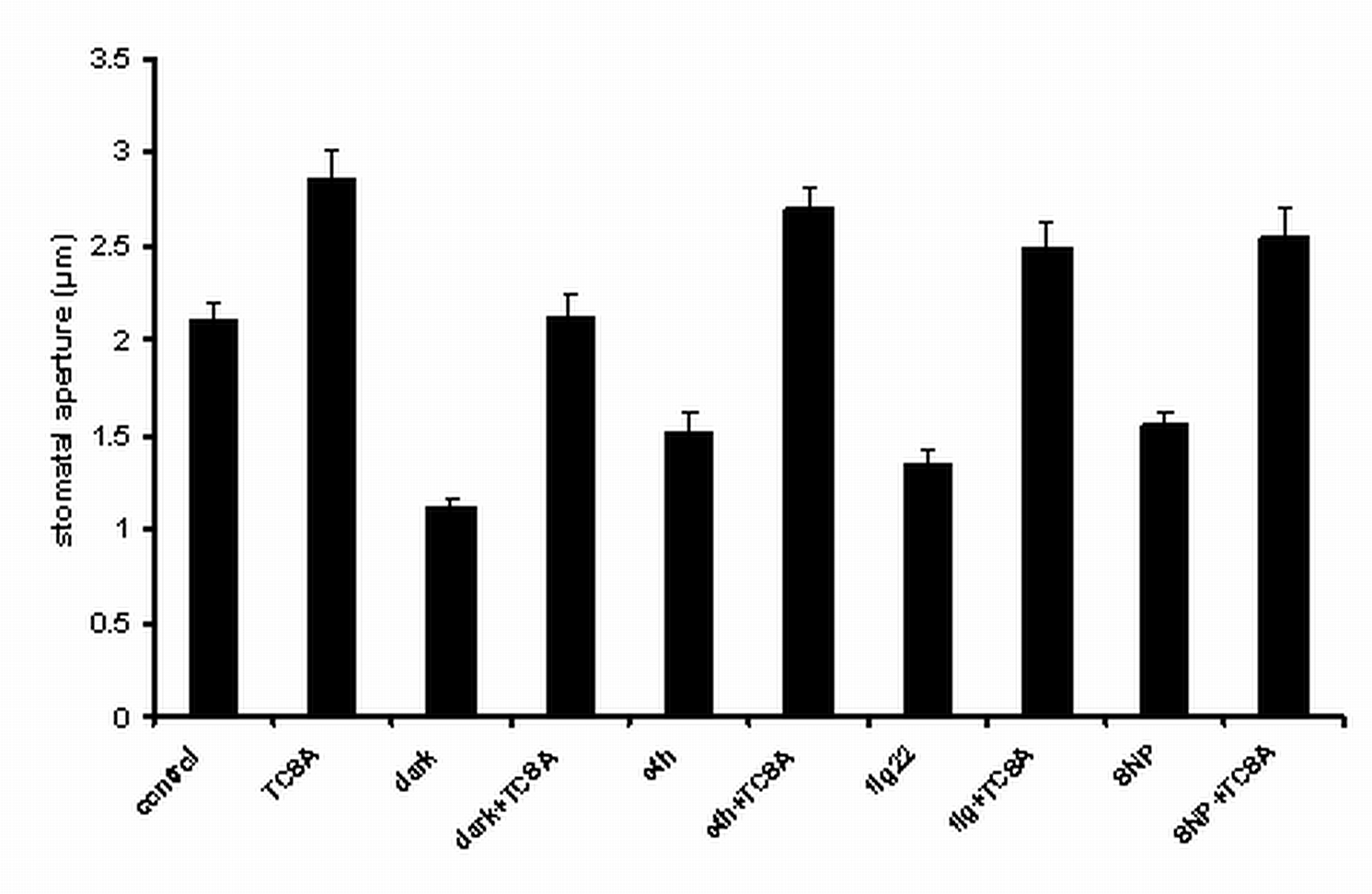

Supplement: Figure S2 — Histidine kinase (HK) activity is required for NO-, dark- and flg22-induced stomatal closure. Effect of the HK inhibitor 3,3′,4′,5-tetrachlorosalicylanilide (TCSA) on stomatal closure in wild type Arabidopsis (Col-0). Arabidopsis leaves were incubated in stomatal opening buffer for 2.5 h followed by treatment for 15 min with 10 μM of TCSA prior to exposure to darkness, ethephon (eth, 100 μM), flg22 (100nM) or SNP (50 μM) for 2.5 h. Control, buffer alone. Data are expressed as mean +/− S.E. from 3 independent experiments (n = 60 guard cells). (0.61 MB TIF) [file pone.0002491.s002.tif]
