# Supplementary material for: The Histidine Kinase AHK5 Integrates Endogenous and Environmental Signals in Arabidopsis Guard Cells
Source: PLoS One. 2008 Jun 18;3(6):e2491. doi: 10.1371/journal.pone.0002491 (PMC2424244; doi:10.1371/journal.pone.0002491)
Supplement: Data S1 — (0.02 MB DOC) [file pone.0002491.s003.doc]

**Primer sequences used for PCR in Figure 2.**

(all in 5’ – 3’ orientation)

1: GTCAGGAGTAGTGGGAATGGCTGAG

2: CTCAGTGCAAATACTGTTGC

3: AAATGCTTGAGGCGAGTACTGA

4: GTTGGCCTCCCATTAACTCAACCAG

5: TGTTTACAACAAAGCTGGCG

6: CAAATTGGTGAGGATCTGCC

7: GCTGTGACGTATGGGACACTGG

8: CGGTCCAAGAGCTTACTCAG

9: AAAGTATCTCAGCCATTCCC

10: TCATGAGACATCGTCGCTAGC

ACT2F: CTGCTCAATCTCATCTTCTTCC

ACT2R: GACCTGCCTCATCATACTCG

EF1FOR: AGCACGCTCTTCTTGCTTTC

EF1REV: GGGTTGTATCCGACCTTCTTC
